# Nonvolatile and Volatile Memory Fusion of Antiferroelectric-like Hafnium–Zirconium Oxide for Multi-Bit Access and Endurance >10^12^ Cycles by Alternating Polarity Cycling Recovery and Spatially Resolved Evolution

**DOI:** 10.1021/acsami.4c14132

**Published:** 2025-02-20

**Authors:** Cheng-Hong Liu, Kuo-Yu Hsiang, Zhi-Xian Li, Fu-Sheng Chang, Zhao-Feng Lou, Jia-Yang Lee, Chee Wee Liu, Pin Su, Tuo-Hung Hou, Min-Hung Lee

**Affiliations:** † Program for Semiconductor Devices, Materials, and Hetero-integration, Graduate School of Advanced Technology, National Taiwan University, Taipei 106319, Taiwan; ‡ Institute of Electronics, National Yang Ming Chiao Tung University, Hsinchu 300, Taiwan; ∥ Institute of Electro-Optical Engineering, National Taiwan Normal University, Taipei 11677, Taiwan; § Graduate Institute of Electronics Engineering, National Taiwan University, Taipei 10617, Taiwan; # Institute of Applied Mechanics, National Taiwan University, Taipei 10617, Taiwan

**Keywords:** ferroelectric (FE), antiferroelectric-like, antiferroelectric (AFE), endurance, recovery, nonvolatile memory (NVM), volatile memory (VM)

## Abstract

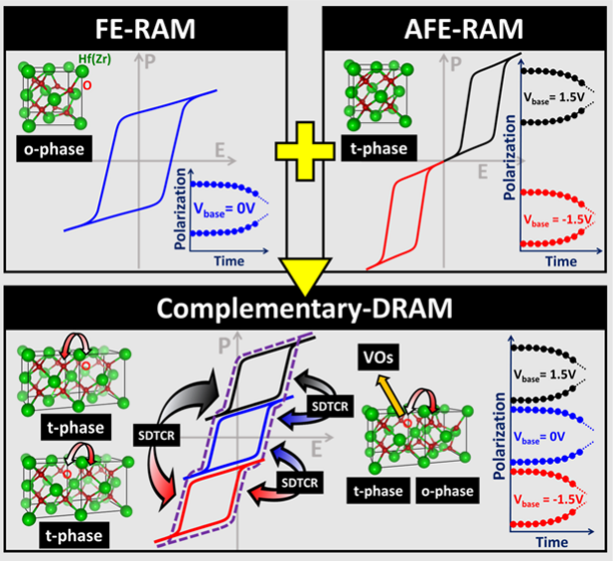

The fusion of volatile and nonvolatile memory within a complementary-dynamic random-access memory (C-DRAM) in one cell is proposed with antiferroelectric-like hafnium–zirconium oxide with dual function characteristics of DRAM and storage class memory in the memory hierarchy. Atomic-resolution spherical aberration-corrected scanning transmission electron microscopy is employed to directly reveal the insights of phase evolution by utilizing diffractograms to determine lattice parameters. Storage-data-transfer-cycling-recovery with an alternating access scheme is introduced due to the characteristic of independent domains to exhibit multilevel states. Remanent polarization (*P*_r_) can be restored, and 34 periods (3 × 10^10^ cycles/period) to accumulate 1.02 × 10^12^ switching cycles are demonstrated. The proposed method is effective in prolonging the endurance and evaluating the spatially resolved evolution of polarization.

## Introduction

The traditional von Neumann architecture limited latency and energy consumption due to the abundance of data transmission in the AI era. Emerging memory technologies have been reported for high density, low power consumption, high access speed, latency improvement, and scalability, such as resistive random-access memory (RRAM), phase change memory (PCM), magnetoresistive random-access memory (MRAM), and ferroelectric random-access memory (FE-RAM).^1−12^ Recently, the hafnium oxide-based ferroelectric device exhibits versatility and attracts a lot of attention for applications such as electrostatic for energy storage, pyroelectric for energy harvesting, and electrocaloric for cooling.^13−21^ Generally, hafnium oxide-based FE-RAM has remarkable nonvolatility characteristics, applicable access speed, and low energy consumption.^22,23^ To further improve the speed and endurance, a hafnium–zirconium oxide (HZO) antiferroelectric (AFE) device would be employed, and several publications reported characteristics comparable to dynamic random-access memory (DRAM).^22−27^ In addition, the recovery techniques of ferroelectric (FE) and AFE have been widely investigated to prolong endurance.^26−30^

**Figure 1:**
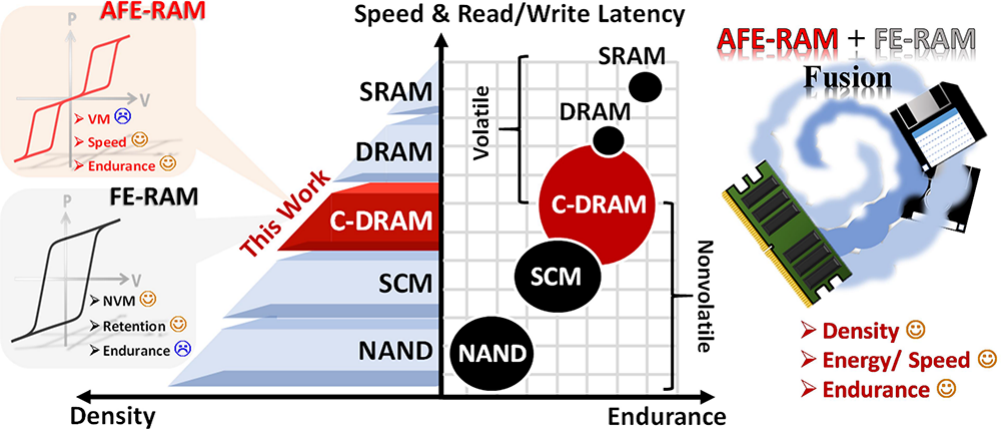
Traditional von Neumann architecture faces latency and energy consumption challenges from data transmission. In this work, the fusion of volatile AFE-RAM and nonvolatile FE-RAM within a single cell is proposed with antiferroelectric-like zirconium-doped hafnium oxide.

In this letter, we propose the fusion of volatile and nonvolatile memory within a complementary-DRAM (C-DRAM) in one cell with antiferroelectric-like HZO. The annular bright-field (ABF) images from spherical aberration-corrected scanning transmission electron microscopy (Cs-corrected STEM) examine the microscopic mechanisms with the different states of phase, crystallinity, lattice constant, and plane orientation from the diffractogram of the device.^31−34^ The proposal eliminates data transmission latency and energy consumption between the main memory system and SCM. Furthermore, the development of the access scheme of storage-data-transfer-cycling-recovery (SDTCR) for C-DRAM application pursues the idea that AFE-RAM and FE-RAM are simultaneous toward endurance immunity to integrate the memory hierarchy of von Neumann architecture, as shown in Figure 1.

## Results and Discussion

### Independent Polarity Access for Antiferroelectric

**Figure 2:**
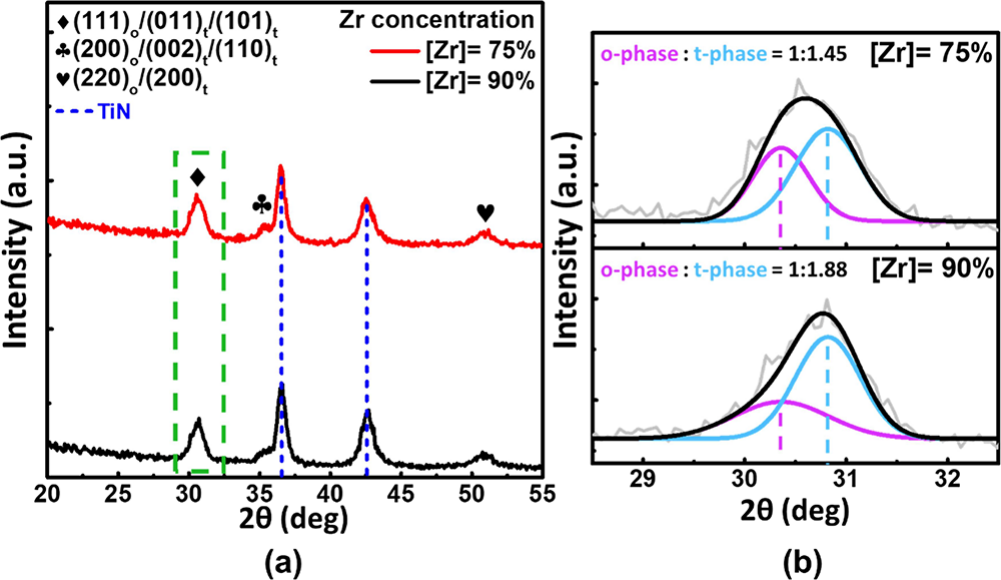
(a) Grazing-angle incidence X-ray diffraction spectra with a 2θ range of 20–55° of [Zr] = 75 and 90%, respectively. (b) Relative phase fraction ratio of the o-phase and t-phase corresponding to the zirconium concentration.

Curie temperature determines the characteristics of FE to AFE in Hf_1–*x*_Zr_*x*_O_2_ (HZO) and can be adjusted by the zirconium concentration in the Zr:HfO_2_ system.^18,35^ The HZO exhibits a remarkable single and double hysteresis loop for FE and AFE characteristics with [Zr] = 50 and 90%, respectively. To understand the crystal structures of HfO_2_–ZrO_2_ films, grazing incidence X-ray diffraction (GIXRD) experiments were applied to identify the presence of orthorhombic and tetragonal phases. With increasing concentration of zirconium, the evolution of the orthorhombic to the tetragonal phase change of the films can be observed, as depicted in Figure 2a. The diffraction peaks near a 2θ of 30.5° are attributed to the overlapped fluorite-based orthorhombic (111) (o(111)) and tetragonal (011) (t(011)) peaks, which is a useful way to discern polar and nonpolar fraction.^36^ Nanobeam diffraction (NBD) patterns were used to extract the d-spacing and determine a strain of 0.7% for the tetragonal and orthorhombic phases, as shown in Figure S1. Consequently, the shifted peak positions of orthorhombic and tetragonal phases are determined to be at 30.4 and 30.8° for the deconvolution of the GIXRD spectra, representing the o(111) and t(101) peaks, respectively. Note that the relaxation peak positions of o(111) and t(101) are 30.1 and 30.6°, respectively, based on the lattice parameters of the HZO thin film.^37^ The results of the relative ratio are presented in Figure 2b with o:t = 1:1.45 and 1:1.88 for Hf_0.25_Zr_0.75_O_2_ and Hf_0.1_Zr_0.9_O_2_, respectively.

**Figure 3:**
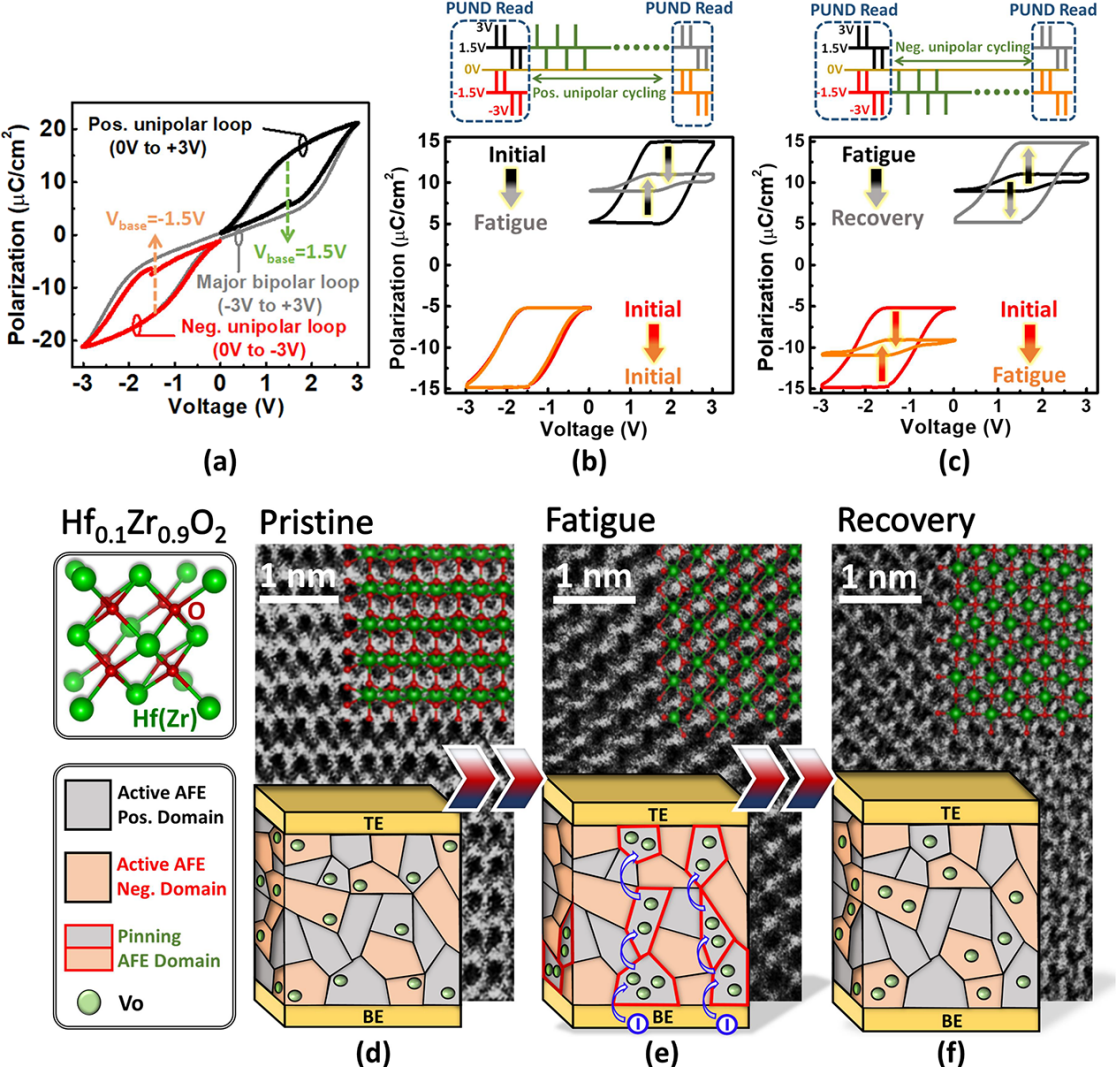
*P*–*V* of AFE Hf_0.1_Zr_0.9_O_2_ for the (a) pristine state, (b) fatigue state after positive unipolar cycling, and (c) recovery of the fatigue state by the previous opposite polarity cycling. ABF-STEM images of the (d) pristine, (e) fatigued, and (f) recovery states. The accumulation or redistribution of VOs induces an unbalanced Zr–O bond length and results in atomic structural displacements in HZO.

The independent polarity of AFE is a unique characteristic due to the double hysteresis loop and single hysteresis loop of polarization–voltage (*P*–*V*) for the bipolar and unipolar sweeps, as shown in Figure 3a. Because of zero remnant polarization for AFE, the data storage would disappear without voltage supporting. This means that the single hysteresis loop of AFE with the base voltage (*V*_base_) has the capability for data storage, which are 1.5 and −1.5 V with the positive and negative unipolar sweep, respectively.^23,26,27^ The *P*–*V* characteristic with the positive-up–negative-down (PUND) procedure shows significant degradation only for the positive loop after positive unipolar cycling. However, the negative loop remains unchanged as the initial state, as shown in Figure 3b, and the corresponding pulse sequences are shown below. The independent polarity operation can be realized in AFE. In addition, the leakage current density (*J*) presents a positive correlation, which increases with the same polarity cycling, and the opposite polarity *J* remains almost unchanged, as shown in Figure S2a,b.

Opposite polarity cycling (negative unipolar cycling) is subsequently employed to determine the fatigued polarity, completely restoring the original fatigued polarity loop to its initial state from degradation, as shown in Figure 3c. The corresponding pulse sequences are shown below. This opposite polarity–cycling recovery (OPCR) can be applied for alternating polarity cycling operations.^26,27^Figure S2c shows the same magnitude of Δ*P*_r_ and *J* via OPCR for the first and third positive unipolar cycling periods to indicate sufficient recovery. As a result, the domains of AFE can be divided into positive and negative polarities independently of the tetragonal phase (t-phase).^26,27^ Furthermore, oxygen vacancies (VOs) accumulate in the corresponding domains, leading to leakage path formation and domain pinning for fatigue. The aggregation of VOs induces an unbalanced Zr–O bond length resulting in atomic structural displacements in HZO and revealing the fatigue state, as shown in Figure 3e.^38^ The result revealed from electrical characteristic (Figure 3c) implies the VO redistribution from the occupied domain to the vacant domain for the original domain depinning, and the recovery of the annular bright-field scanning transmission electron microscopy (ABF-STEM) image observes that the atomic structure of HfZrO_2_ returns to a stable arrangement, as shown in Figure 3f. In Figure 3d, the random distribution of VO for the pristine state indicates that the Hf and Zr atoms locate a stable arrangement by local observation of the ABF-STEM image. Due to the absence of VOs, the Zr–O bond length remains consistent, resulting in the t-phase structure being well matched to pristine and recovered grains in Figure 3d,f.

### Duality of Volatile and Nonvolatile for Antiferroelectric-like

**Figure 4:**
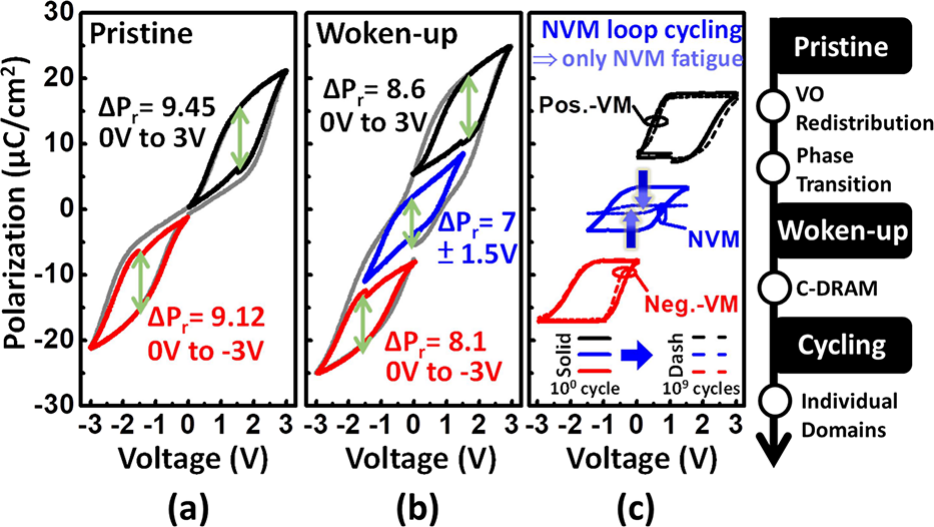
*P*–*V* of AFE-like for (a) pristine, (b) woken-up, and (c) fatigued states. Significant degradation for fatigue is observed only in NVM cycling. AFE-like domains are revealed with individual domains, which are the o-phase and t-phase (positive and negative polarities) for independent switching.

The preceding AFE recognized the independent polarity for negative and positive loops and implied volatile data storage without *V*_base_. To meet the nonvolatile memory application, the AFE-like is adopted by [Zr] = 75% of HZO. The *P*–*V* of the pristine state for AFE-like exhibits typical AFE double-loop and single-loop characteristics for bipolar and unipolar operation, respectively, as shown in Figure 4a. Subsequently, a significant enhancement in *P*_r_ compared to the pristine state with the wake-up procedure is shown in Figure 4b. This is contributed by the redistribution of VOs and the phase transition of partial t-phase transitions to the o-phase after the wake-up procedure. The *P*–*V* characteristics of AFE-like are subdivided into three loops and serve as multilevel memory for the dual functionalities of DRAM. They are positive and negative unipolar loops with *V*_base_ = 1 and −1 V for volatile memory (VM) and a minor bipolar loop for nonvolatile memory (NVM) with *V*_base_ = 0 V. For fatigue after minor bipolar cycling, significant degradation is observed only in the NVM loop; however, the other two nonoperation VM loops remain unchanged, as shown in Figure 4c. Positive and negative unipolar VMs in AFE have been investigated with switching independently of existing domains. In summary, the AFE-like domain is divided into independent polarities and dominated by the o-phase and positive/negative t-phase, i.e., minor bipolar NVM and positive/negative unipolar VM. The multilevel states (minor bipolar loop and positive and negative unipolar loops) for the AFE-like exhibit independent polarities on fatigue, as shown in Figure S3 in the Supporting Information.

**Figure 5:**
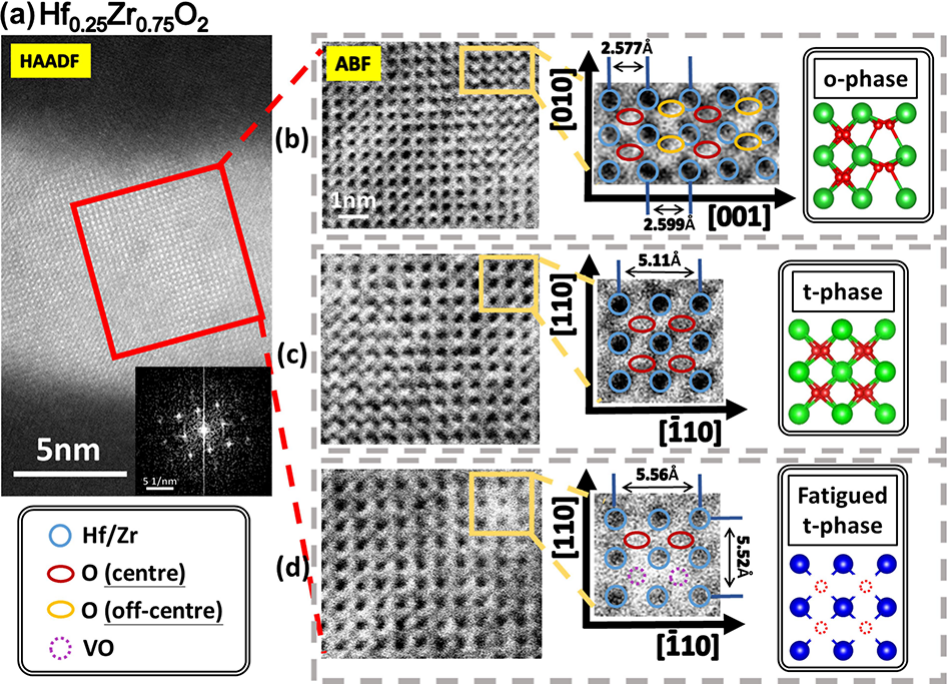
(a) Atomic-resolution HAADF-STEM image for Hf_0.25_Zr_0.75_O_2_. (b–d) The magnified view of the ABF-STEM images represents the o-phase, t-phase, and fatigued t-phase, respectively. Note that the sample was observed in ex situ conditions after positive unipolar cycling.

To further understand the presence of the o-phase and t-phase structure, the overall atomic-resolution high-angle annular dark field (HAADF) of Cs-corrected STEM is employed, as shown in Figure 5a.^40,41^ Additionally, the ABF-STEM images are applied to enhance the visibility of oxygen atoms, enabling a clearer visualization and more accurate classification of the phases. Within this view, three discrete atomic sites exhibit various arrangements of oxygen atoms, indicating the presence of diverse phases. Figure 5b shows the distribution of oxygen atomic positions off the centrosymmetric position and is consistent with the o-phase characteristics. In contrast, all of the oxygen atoms are in central positions, implying the t-phase with no residual polarization, as shown in Figure 5c. In addition, a portion of oxygen atoms migrate toward the periphery, resulting in an increase in lattice constants attributed to the formation of VOs, as shown in Figure 5d.^34,40,41^ Combining the information on lattice constant and the plane orientation, these phases can be identified as the o-phase, t-phase, and fatigued t-phase. Note that the sample was observed in ex situ conditions after positive unipolar cycling.

### Model for VO Redistribution by Alternating Polarity Cycling

**Figure 6:**
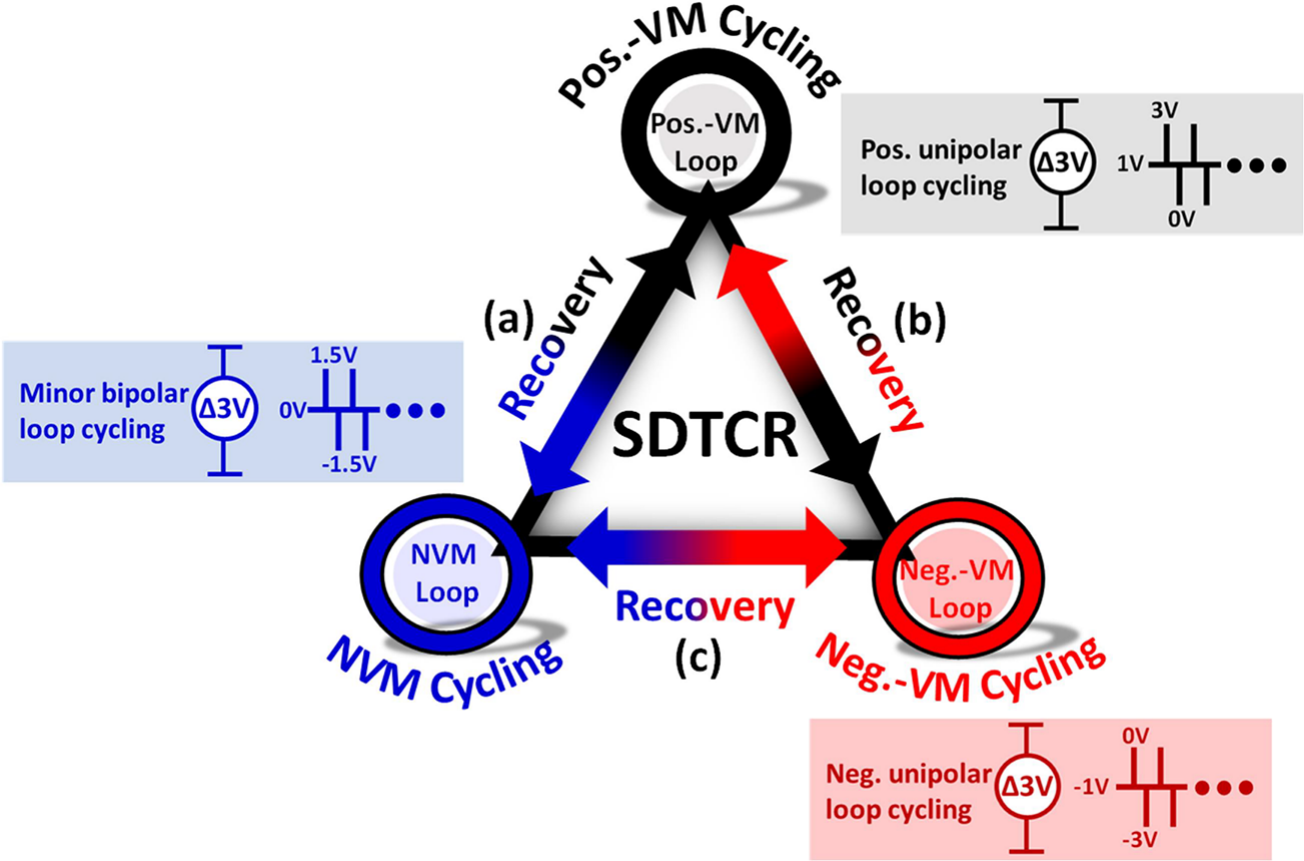
Three kinds of procedures for endurance characteristics with alternating cycles: (a) minor bipolar loop ↔ positive unipolar loop, (b) minor bipolar loop ↔ negative unipolar loop, and (c) positive unipolar loop ↔ negative unipolar loop, with the corresponding pulse sequence of cycling. Note that each loop can be operated independently.

**Figure 7:**
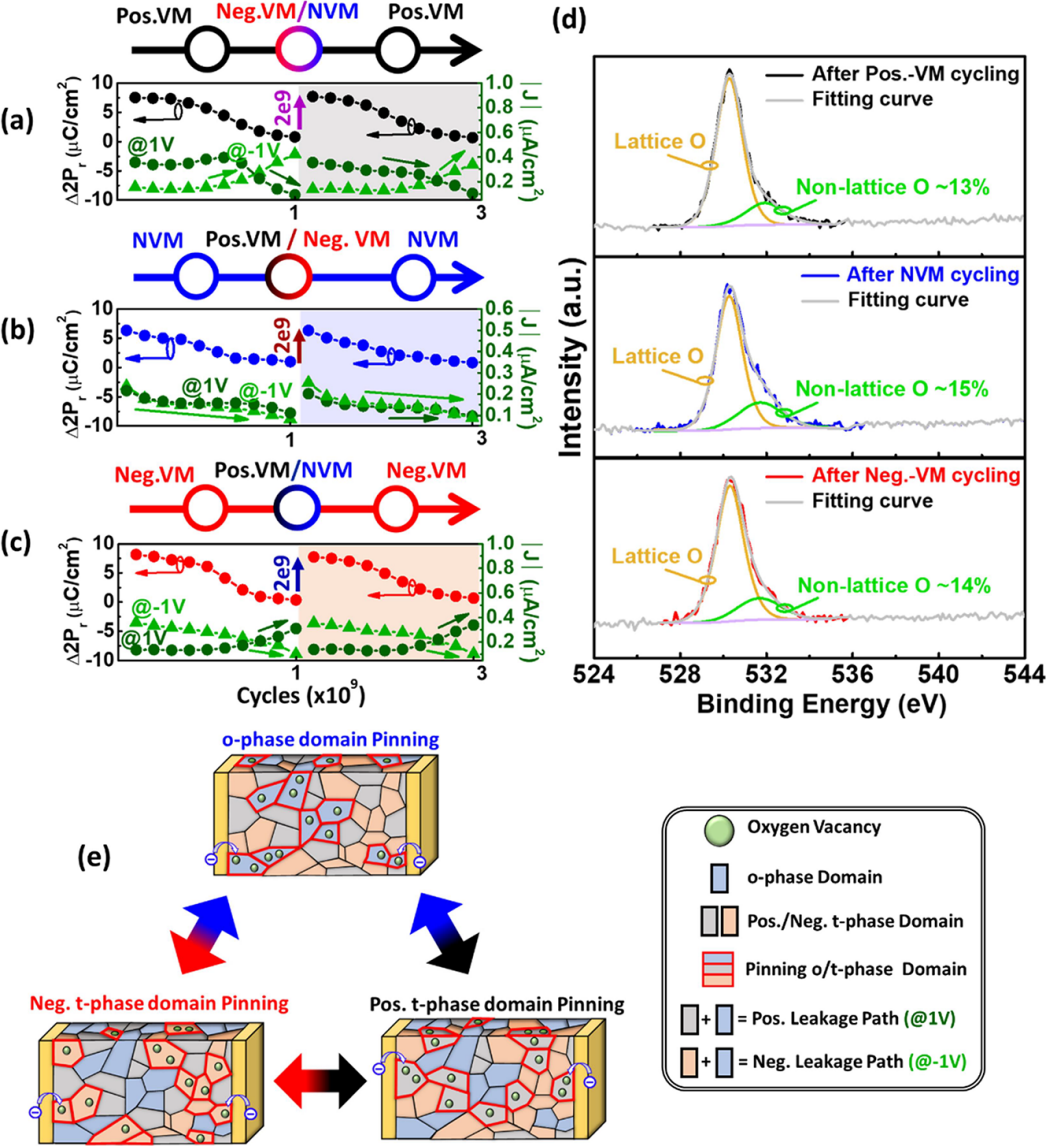
Proposed model illustrates the VO redistribution, *J* and 2*P*_r_ with cycling for (a) positive unipolar cycling, (b) minor bipolar cycling, and (c) negative unipolar cycling. The accumulation of VOs in the corresponding domain dominates the fatigue and the redistribution of VOs with the other loop cycling for the recovery, which are validated by extracting increment/decrement in *J* for positive and/or negative polarities. VOs accumulate in the operated domains as traps to inhibit electron conduction and suppress *J*. (d) VO magnitude is confirmed to be a nonsignificant difference after pos.-VM, NVM, and neg.-VM cycling by XPS. (e) The schematic diagram illustrates VO migration among the o-phase, positive t-phase, and negative t-phase.

Figure 6 illustrates three kinds of procedures for endurance characteristics with alternating cycles: (a) minor bipolar loop ↔ positive unipolar loop, (b) minor bipolar loop ↔ negative unipolar loop, and (c) positive unipolar loop ↔ negative unipolar loop, as well as the pulse sequence shown in the corresponding cycling. With the wake-up procedure, the original t-phase domain of AFE-like transfers to the o-phase domain, and the domain configuration schematic of AFE-like is depicted in Figure 7e. Figure 7a–c also demonstrates the recovery characteristics for the multilevel states of the proposed AFE-like. The fatigue and recovery mechanisms are investigated by extracting Δ*P*_r_ and *J* with cycling, and VO is extracted by oxygen 1s XPS spectra after NVM and VM cycling. The XPS results are a nonsignificant difference, which indicates that the total VO magnitude remains similar to the cycling operations with the redistribution, as shown in Figure 7d. The 13–15% of VO is reasonable for the HfO_2_-based material and agrees with previous works.^42−44^ Therefore, the VO redistribution in the domain dominates the fatigue and recovery mechanisms. First, the fatigue mechanism with cycling is due to the accumulation of VOs and leads to domain pinning.^26,27,29,39^ The bipolar *J* decreases with minor bipolar loop cycling due to the increment of the o-phase domains in the AFE-like, which increases the interval between domains and reduces the probability of trap-assisted tunneling (TAT). However, the unipolar *J* decreases and increases with unipolar loop cycling of the same and opposite polarity, respectively. This result implies that VOs accumulate in the operated domains as traps to inhibit electron conduction and suppress *J*. In contrast, the nonoperated domains lack VOs, and the *J* increases slightly. As a result, the accumulation of VOs leads to a decrease in *J*, which is a clear difference from the mechanisms of AFE and FE.^26,27,29^ Note that the o-phase cannot separate unipolar domains, which is distinctly different from that of the t-phase. Hence, the extracted leakage paths for positive and negative polarities are divided into the positive t-phase domain and o-phase domain, as well as the negative t-phase domain and o-phase domain. For the recovery mechanism, the accumulation of VOs in the corresponding domain dominates the fatigue and the redistribution of VOs with opposite polarity loop cycling for the recovery in Figure 7a–c. The top figure of Figure 7b describes the recovery of the fatigued minor bipolar loop, where the recovery procedure by positive and/or negative unipolar loop cycling redistributes the accumulated VOs of the o-phase domain to the t-phase domains with unipolar cycling. Note that the accumulation and redistribution of VOs with cycling are validated by extracting the increment/decrement in *J* for positive and/or negative polarities. In addition, the top figure of Figure 7a,c describes the fatigue and recovery procedure between positive and negative unipolar loops, and the result is comparable with AFE.^26,27^ Note that the positive and negative unipolar loops can also attain the recovery strategy with a minor bipolar loop. In summary, the operation sequences for multilevel states of AFE-like are independent and have the potential for unlimited endurance for DRAM requirements.

### Demonstration of Recovery Scheme for Prolongation Endurance

**Figure 8:**
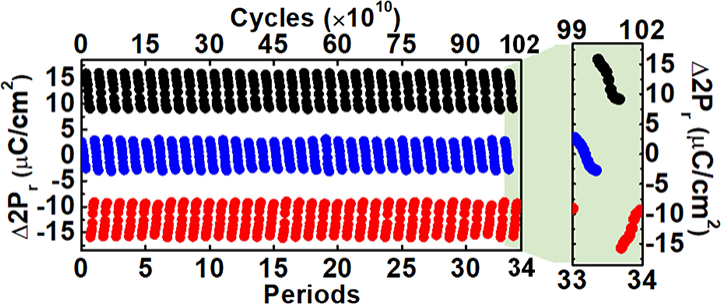
C-DRAM with 34 periods by SDTCR accumulates to the 1.02 × 10^12^ switching cycle. Note that the endurance measurement begins with the NVM loop, followed by the positive VM loop, and then the negative VM loop.

The fusion of volatile and nonvolatile memory within one cell by AFE-like is denoted as C-DRAM. Furthermore, this work proposes the concept of SDTCR. It involves alternating access to short-term storage unipolar loops, i.e., positive/negative VM loops, and long-term storage bipolar loops, i.e., the minor bipolar NVM loop, accomplishing the recovery procedure. To validate the SDTCR concept and the long endurance performance of the hypothesis. Figure 8 demonstrates the multilevel state cycling of a C-DRAM for 34 periods (3 × 10^10^ cycles/period) by SDTCR and accumulating to 1.02 × 10^12^ switching cycles, in which the procedure starts with the NVM loop, followed by the positive VM loop, and then the negative VM loop. Note that the measurement duration is about 7 weeks (1.5 months) for such long endurance testing. The nondegradation and complete restoration of 3-bit multilevel state Δ*P*_r_ were measured to determine the prospect of unlimited endurance by SDTCR. Note that the data retention capability of the NVM loop exhibits excellent characteristics with >10^4^ s, as shown in Figure S4 in the Supporting Information, and the detailed stress cycling of this work and prior works is presented in Figure S5. Compared with the recovery technologies of prior works on FE-RAM and AFE-RAM, the multilevel states of C-DRAM with SDTCR not only simultaneously integrate volatile and nonvolatile memory within a single cell to reduce the recovery voltage but also achieve a recovery time ratio of 0% (*t*_recovery_/*t*_period_), indicating no extra time to spend for the recovery procedure, as shown in Table 1. Note that the frequency is 0.25 MHz to confirm almost complete dipole switching, suggesting a relatively rigorous stress condition compared to prior works.^25−27^

**Table 1:** Comparison of the Recovery Technologies in Prior Works^a^

Fe-based RAM	C-DRAM	FE-RAM	FE-RAM	FE-RAM	AFE-RAM
materials	Hf_0.25_Zr_0.75_O_2_	Hf_0.5_Zr0_0.5_O_2_	Hf_0.5_Zr0_0.5_O_2_	Hf_0.5_Zr0_0.5_O_2_	Hf_0.1_Zr_0.9_O_2_
nonvolatile	√	√	√	√	×
volatile	√	×	×	×	√
multi-bit	√	√	×	×	√
cycling Δ*V* (V)	3	4	4	3.5	3
recovery Δ*V* (V)	3	4	8	7	3
frequency (MHz)	0.25 MHz	0.25 MHz	1 MHz	5 MHz	0.25 MHz
recovery method	SDTCR	AFCR	high E-field	high E-field	OPCR
recovery time (%)	0%	0%	∼0.1%	1e-6%	0%
cumulative cycles	1.02 × 10^12^	10^12^	10^9^	10^12^	10^12^
references	this work	(28)	(29)	(30)	(26) and (27)

a This work provides the 3-bit MLC of C-DRAM with SDTCR, which not only integrates VM and NVM within a single cell to reduce the *V*_recovery_ but also achieves a *t*_recovery_ ratio of 0%.

## Summary

In this work, the fusion of volatile and nonvolatile memory within a C-DRAM in one cell is proposed to improve the speed and energy consumption of data transfer between the main memory and storage class memory. The spatially resolved analysis of domain phases validates the o-phase, t-phase, and fatigued t-phase in the AFE-like HZO thin film with [Zr] = 75%. Furthermore, the multilevel states of C-DRAM combined with SDTCR lead to unlimited endurance breakthrough in current memory architectures.

## Experimental Methods

### Device Fabrication

A metal–insulator–metal (MIM) structure was fabricated for the capacitors, in which 10 nm-thick Hf_1–*x*_Zr_*x*_O_2_ (HZO) was employed by atomic-layer deposition (ALD) at 250 °C. The HZO was controlled by the supercycle of HfO_2_ and ZrO_2_ with ratios of 1:3 and 1:9 for AFE-like and AFE, respectively. The precursors of the former and latter are tetrakis(dimethylamino)hafnium (TDMA-Hf) and tetrakis(dimethylamino)zirconium (TDMA-Zr), respectively. The 50 nm-thick TiN was employed as the top and bottom electrodes by a sputtering system. Subsequently, HZO crystallization was performed by rapid thermal annealing (RTA) at 600 °C in ambient Ar for 60 s. The wake-up procedure was conducted at 3 V and for 10^4^ cycles. Note that the size of the top electrode is 100 μm × 100 μm for the capacitors, which is also identical to an active area of HZO.

### Electrical Measurements

A semiconductor parameter analyzer, Keysight B1500A, was utilized with modules B1517A for current density–voltage (*J*–*V*), B1530A for positive-up–negative-down (PUND), and B1525A for a pulse generator to characterize endurance and retention. The endurance cycling was performed with 0.25 MHz (pulse width of 1 μs) and a rise/fall time of 20 ns. The polarization–voltage (*P*–*V*) was measured by a ferroelectric tester Radiant Premier II with a triangular waveform with a frequency of 10 kHz.

### GIXRD Measurement

To evaluate the crystalline structure and crystallinity of the nanoscale HZO thin films, the patterns were acquired using a high-power X-ray diffractometer with a Cu Kα X-ray source (λ = 0.154 nm), the incidence angle (ω) was fixed at 1°, and the 2θ scan was conducted between 20 and 80° by a step of 0.06°.

### XPS Measurement

The ratio of nonlattice oxygen (VOs) in the Hf_0.25_Zr_0.75_O_2_ films was determined using XPS with a monochromatic Al Kα source at 1486.6 eV (K-Alpha+ XPS Spectrometer). Additionally, the high-resolution O 1s spectrum was deconvoluted into three synthetic peaks centered at 530.3 and 531.8 eV, corresponding to lattice oxygen (O^2–^) and oxygen vacancies (VOs), respectively.

## Supporting Information Available

A detailed description of the NBD diffraction by considering stress-induced peak shift, the current density of AFE with unipolar switching, demonstration of the independency of AFE-like, data retention characteristic, and the available switching 2*P*_r_ versus pulse width (PDF)
