# Supplementary material for: Nonvolatile and Volatile Memory Fusion of Antiferroelectric-like Hafnium–Zirconium Oxide for Multi-Bit Access and Endurance >1012 Cycles by Alternating Polarity Cycling Recovery and Spatially Resolved Evolution
Source: ACS Appl Mater Interfaces. 2025 Feb 20;17(9):14342–9. doi: 10.1021/acsami.4c14132 (PMC11891857; doi:10.1021/acsami.4c14132)
Supplement: Supplementary file 1 — am4c14132_si_001.pdf [file am4c14132_si_001.pdf]

“Supporting Information

Nonvolatile and Volatile Memory Fusion of Antiferroelectric-like Hafnium-Zirconium Oxide for Multi-Bit Access and Endurance > 10^{12} Cycles by Alternating Polarity Cycling Recovery and Spatially Resolved Evolution”

Cheng-Hong Liu,[†, ¶] *Kuo-Yu Hsiang,*[†, ‡, ¶] *Zhi-Xian Li,*[∥] *Fu-Sheng Chang,*[†, §] *Zhao-Feng Lou,*[†] *Jia-Yang Lee,*[†] *Chee Wee Liu,*[†, §] *Pin Su,*[‡] *Tuo-Hung Hou,*[‡] *and Min-Hung Lee*[†, §, #, *]

[†]Program for Semiconductor Devices, Materials, and Hetero-integration, Graduate School of Advanced Technology, National Taiwan University, Taipei 106319, Taiwan

[‡]Institute of Electronics, National Yang Ming Chiao Tung University, Hsinchu 300, Taiwan

[∥]Institute of Electro-Optical Engineering, National Taiwan Normal University, Taipei 11677, Taiwan

[§]Graduate Institute of Electronics Engineering, National Taiwan University, Taipei 10617, Taiwan

[#]Institute of Applied Mechanics, National Taiwan University, Taipei 10617, Taiwan

[*]Email: minhunglee@ntu.edu.tw

Considering stress-induced peak shift by NBD diffraction

The diffraction pattern of $Hf_{0.25}Zr_{0.75}O_2$ was employed to extract the d-spacing and determine the strain 0.7% for the tetragonal and orthorhombic phases by nanobeam diffraction (NBD) as shown in **Figure S1**. The FFT diffraction is also reconstructed according to **Figure 3d** for $Hf_{0.1}Zr_{0.9}O_2$ to validate the d-spacing of the tetragonal phase since the relatively low ratio of the orthorhombic phase is difficult to observe.

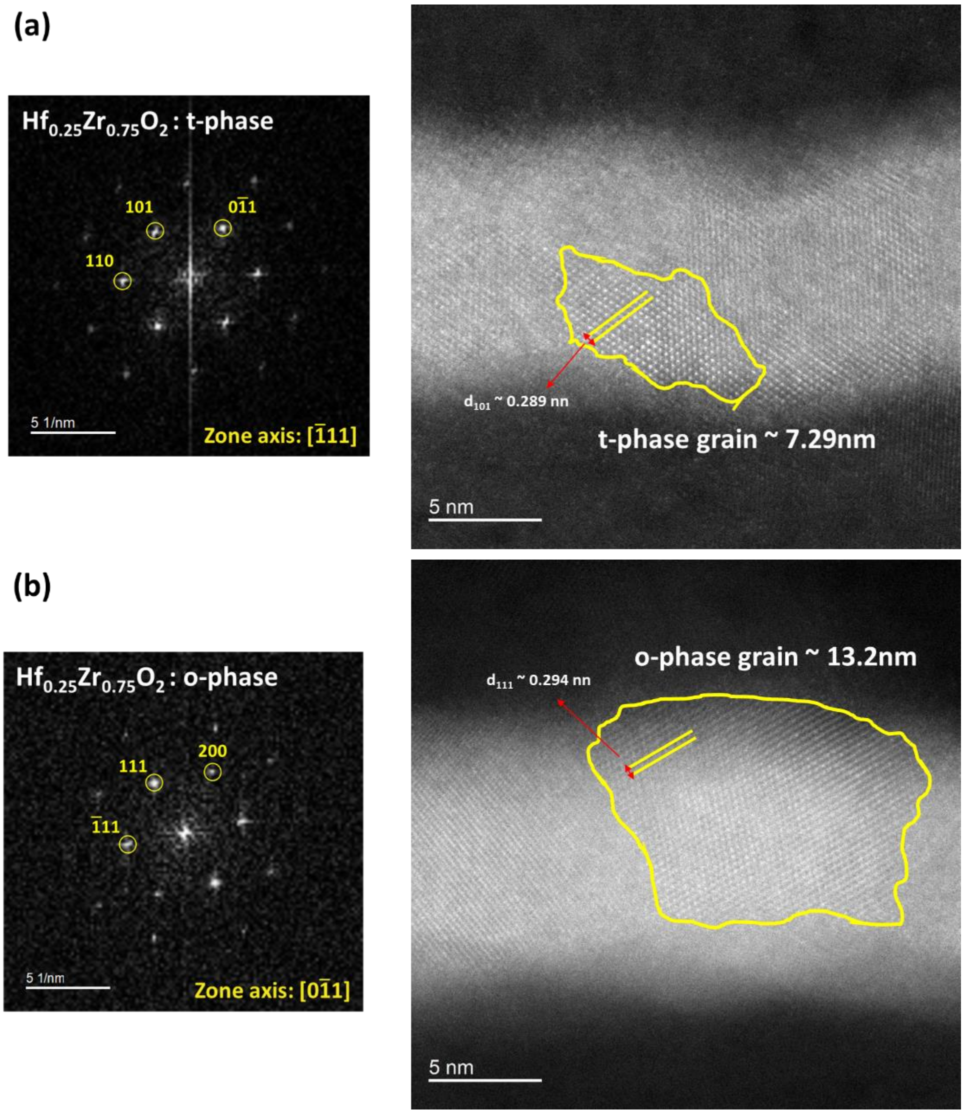

Figure S1. Nanobeam diffraction patterns and STEM-HAADF images of $Hf_{0.25}Zr_{0.75}O_2$ for **(a)** the tetragonal phase and **(b)** the orthorhombic phase.

Current Density of AFE with Unipolar Switching

The current density (J) response of AFE with the positive and negative unipolar operation shows an increase after cycling on the same polarity, but J remains almost unchanged for opposite polarity cycling. **Figure S2a**,**b** shows the cycling of the AFE for the positive and negative unipolar loops, respectively. The J also indicates the same magnitude of ΔP_r and J via Opposite Polarity Cycling Recovery (OPCR) for the 1st and 3rd positive unipolar cycling periods to validate the sufficient recovery in **Figure S2c**.[1,2]

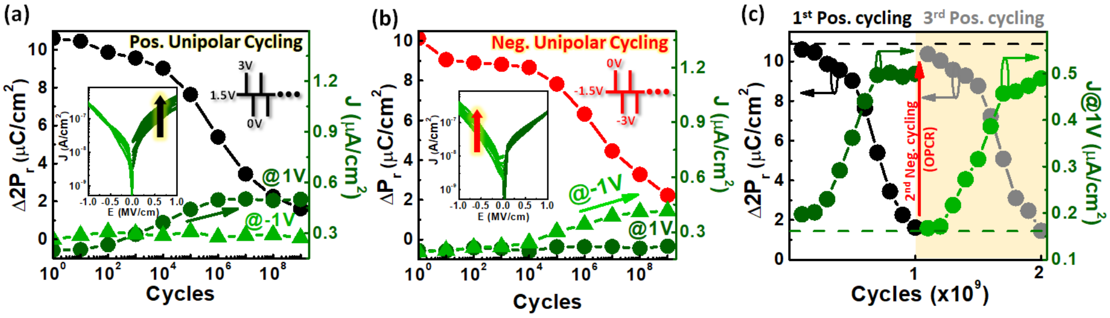

Figure S2. ΔP_r and J of the AFE with **(a)** positive and **(b)** negative unipolar cycling. **(c)** The same magnitude of ΔP_r and J via Opposite Polarity Cycling Recovery (OPCR) for the 1st and 3rd positive unipolar cycling periods to validate the sufficient recovery.

Three Independent Loops of AFE-like

AFE-like exhibits three independent loops with individual switching ranges. The cycling voltage range is shown in **Figure S3a**. **Figure S3b-d** shows the cycling of the AFE-like for the minor bipolar loop, positive and negative unipolar loops, respectively, and the corresponding 3 loops. Note that the maximum magnitude of the write voltage of the three loops (minor bipolar loop, positive and negative unipolar loops) is set at 3 V, and the V_{base} for the positive and negative unipolar loops are reduced to 1 V and -1 V, respectively, which reduces energy consumption compared to AFE. **Figure S3b-d** shows significant degradation in a single loop; however, the other non-operated loops maintain the initial state. This indicates independence for three loops for the AFE-like and is comparable with FE and AFE.[1-3]

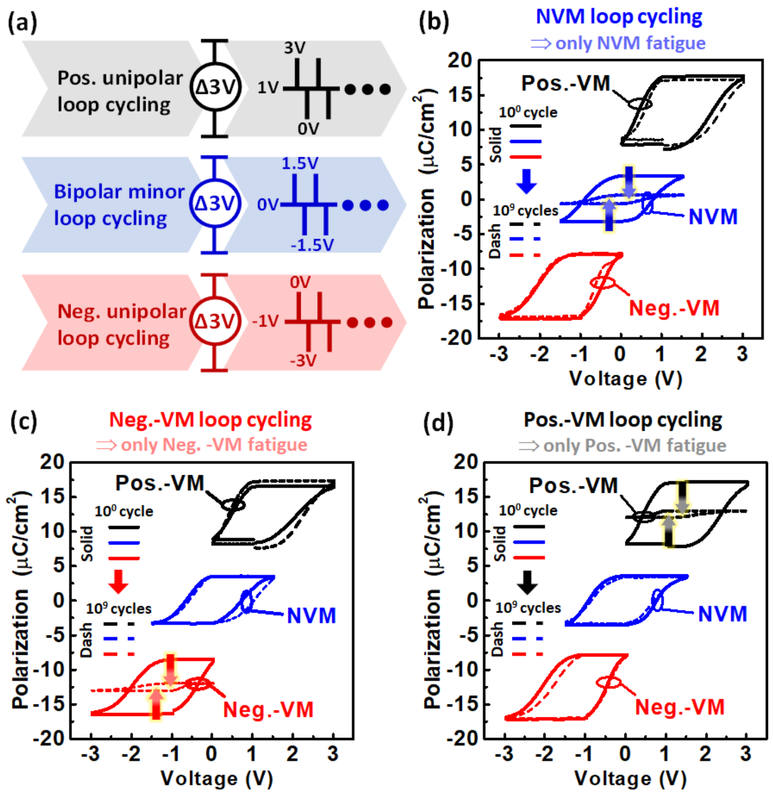

Figure S3. (a) Corresponding input stimulus waveform for three loops for the AFE-like. The cycling for **(b)** bipolar minor loop, **(c)** positive, and **(d)** negative unipolar loops.

Data Retention Characteristics of NVM Loop

Figure S4a shows the corresponding pulse sequence for retention characteristics. **Figure S4b,c** shows the excellent data retention characteristics of the AFE-like with V_{base} = 0V and 1.5V, respectively, which are non-volatile characteristics of minor bipolar switching for the former and unipolar switching for the latter. In the case without V_{base} to support E-field, $2P_r$ will vanish for the unipolar switching, i.e., volatile memory, as shown in **Figure S4c**. The duality of proposed AFE-like presents both nonvolatile characteristics of > 10^4 s and > 10^3 s for minor bipolar and unipolar operation, respectively. The nonvolatile data storage is achieved with minor bipolar switching and goes beyond the requirements of 64ms for DRAM.

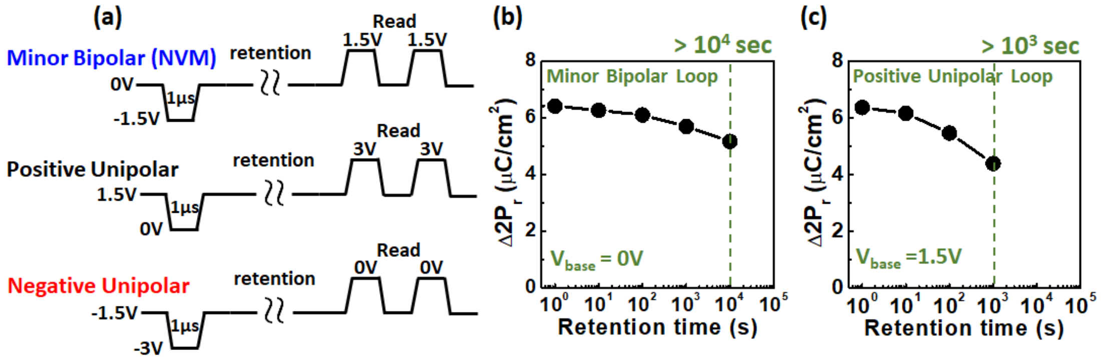

Figure S4. Data retention of **(a)** corresponding pulse sequence, **(b)** minor bipolar switching with V_{base} = 0V, **(c)** unipolar switching with V_{base} = 1.5V. Duality of proposed AFE-like presents both nonvolatile characteristics of > 10^4 s and > 10^3 s for minor bipolar and unipolar operation, respectively. The nonvolatile data storage is

achieved with minor bipolar switching and goes beyond the requirements of 64ms for DRAM.

Available Switching 2P_r vs. Pulse Width

Figure S5a shows the waveform sequence of previous endurance-recovery studies, and this work. In this work, the 0.25MHz and pulse width 1μs were applied for stress cycling. This is a relatively rigorous stress condition as compared to Ref. [5][6]. Since the slower speed and longer pulse width to sufficient dipole switching in **Figure S5b**, severe endurance degradation would occur due to the dipole fully flipping out with fatigue.[4] The SDTCR method is effective in completing recovery to achieve unlimited endurance.

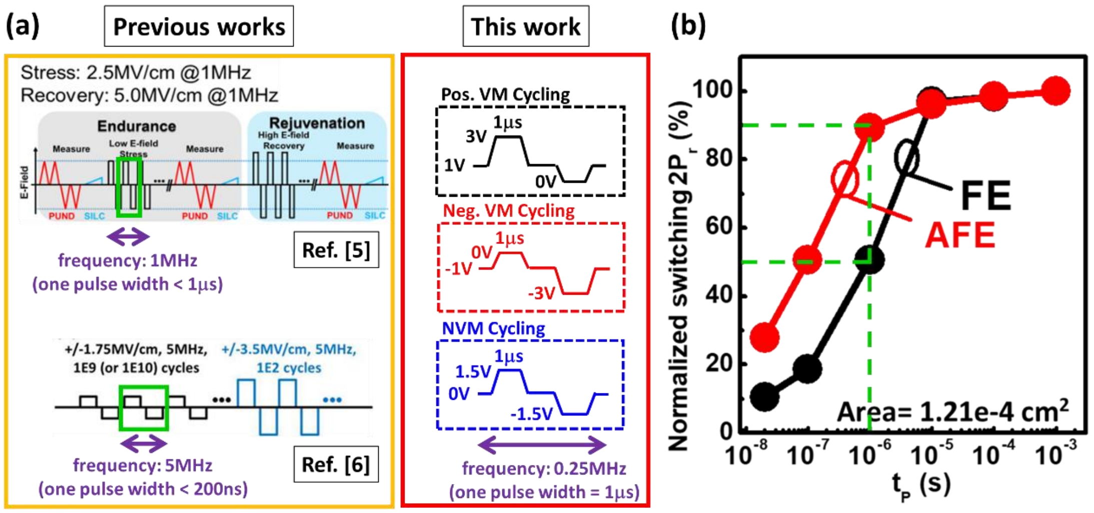

Figure S5. (a) Compared the stress conditions to other endurance-recovery studies and **(b)** Normalized switching 2P_r for FE and AFE capacitors with various programming pulse widths.

References

(1) Hsiang, K.-Y.; Chen, Y.-C.; Chang, F.-S.; Lin, C.-Y.; Liao, C.-Y.; Lou, Z.-F.; Lee, J.-Y.; Ray, W.-C.; Li, Z.-X.; Wang, C.-C.; Tseng, H.-C.; Chen, P.-H.; Tsai, J.-H.; Liao, M. H.; Hou, T.-H.; Liu, C. W.; Huang, P.-T.; Su, P.; Lee, M. H. Novel opposite polarity cycling recovery (OPCR) of HfZrO2 antiferroelectric-RAM with an access scheme toward unlimited endurance. *In 2022 International Electron Devices Meeting (IEDM).* IEEE 2022; pp 32.5.1–32.5.4.

(2) Hsiang, K.-Y.; Lee, J.-Y.; Lou, Z.-F.; Chang, F.-S.; Chen, Y.-C.; Li, Z.-X.; Liao, M.-H.; Liu, C. W.; Hou, T.-H.; Su, P.; Lee, M. H. Fatigue Mechanism of Antiferroelectric $Hf_{0.1}Zr_{0.9}O_2$ Toward Endurance Immunity by Opposite Polarity Cycling Recovery (OPCR) for eDRAM. *IEEE Trans. Electron Devices* 2023, 70, 2142-2146.

(3) Hsiang, K.-Y.; Lee, J.-Y.; Chang, F.-S.; Lou, Z.-F.; Li, Z.-X.; Li, Z.-H.; Chen, J.-H.; Liu, C.; Hou, T.-H.; Lee, M. H. FeRAM Recovery up to 200 Periods with Accumulated Endurance 10^{12} Cycles and an Applicable Array Circuit toward Unlimited eNVM Operations. In *2023 IEEE Symposium on*

VLSI Technology and Circuits (VLSI Technology and Circuits). IEEE 2023; 1-2.

(4) Hsiang, K.-Y.; Liao, C.-Y.; Lin, Y.-Y.; Lou, Z.-F.; Lin, C.-Y.; Lee, J.-Y.; Chang, F.-S.; Li, Z.-X.; Tseng, H.-C.; Wang, C.-C.; Ray, W.-C.; Hou, T.-H.; Chen, T.-C.; Chang, C.-S.; Lee M. H. Correlation between Access Polarization and High Endurance (˜ 10^{12} cycling) of Ferroelectric and Anti-Ferroelectric $HfZrO_2$. In *2022 IEEE International Reliability Physics Symposium (IRPS).* IEEE 2022; pp P 9-1-9-4.

(5) Chang, Y. K.; Liao, P. J.; Yeong, S. H.; Lin, Y.-M.; Lee, J. H.; Lin, C. T.; Yu, Z.; Tsai, W.; McIntyre, P. C. The field-dependence endurance model and its mutual effect in Hf-based ferroelectrics. In *2022 IEEE International Reliability Physics Symposium (IRPS).* IEEE 2022; pp 3A. 1-1-3A. 1-5.

(6) Liao, P. J.; Chang, Y. K.; Lee, Y.-H.; Lin, Y. M.; Yeong, S. H.; Hwang, R. L.; Hou, V.; Nien, C. H.; Lu, R.; Lin, C. T. Characterization of fatigue and its recovery behavior in ferroelectric HfZrO. In *2021 Symposium on VLSI Technology.* IEEE 2021; 1-2.
